# Supplementary material for: Predictors of Turkish individuals’ online shopping adoption: An empirical study on regional difference
Source: PLoS One. 2023 Jul 20;18(7):e0288835. doi: 10.1371/journal.pone.0288835 (PMC10358937; doi:10.1371/journal.pone.0288835)
Supplement: Attachment — Submitted filename: Response to reviewers-27.05.2023.docx [file pone.0288835.s001.docx]

**Dear Editor and Reviewers,**

Thank you very much for your comments concerning our manuscript titled “Determinants of Intimate Partner Controlling Behaviour Targeting Women in Türkiye”. These comments have been very helpful in reviewing and improving our manuscript. We have carefully revising these instructive comments and made corrections that we hope will be approved. The revised parts are highlighted in red in the main paper.

**Additional Editor Comments (if provided):**

Thank you for submitting your paper to PLOSE ONE. We believe that your submission has a merit. However, there are serious issues that need to be addressed by you in order to make your submission suitable for publication. All comments are highlighted in the next section.

The reviewers' comments

**Comment:** The novelty of this paper is presented in a very limited manner. In the introduction, it is important to emphasize the significance of the work and justify its novelty by highlighting its main contributions to the existing literature.

**Response:** Thank you for the comment. Taking this criticism into account, we added the novelty of this paper.

**Comment:** The proposed hypotheses need more theoretical support.

**Response:** Thank you for the comment. Taking this criticism into account, we added additional explanations for the hypotheses.

**Comment:** Please provide a description of the population under study and the sampling technique utilized in the methodology and research design section. Additionally, please provide a rationale for the chosen sample size and sampling technique.

**Response:** Thank you for the comment. Taking this criticism into account, we added the necessary explanations to the Data section.

**Comment:** The authors are advised to dedicate a separate section to highlight the main theoretical and practical implications.

**Response:** Thank you for the comment. Taking this criticism into account, we added the “Theoretical and practical implications” heading.

**Comment:** The literature review is presented in a very limited manner. Thus, it is recommended to extend the literature by including further recent, related and well-established studies. This includes but not limited to:

E-commerce in high uncertainty avoidance cultures: The driving forces of repurchase and word-of-mouth intentions. Doi: https://doi.org/10.1016/j.techsoc.2022.102083

Feasibility assessment for E-commerce: A data collection from developing country (Ethiopia). Doi: <https://doi.org/10.1016/j.mex.2022.101639>

**Response:** Thank you for the comment. Taking this criticism into account, we have cited both the recommended studies and other new studies for literature review.

**Comment:** As e-commerce is highly researched, the discussion part can be broadened a bit more with more relevant research studies.

**Response:** Thank you for the comment. Taking this criticism into account, we revised the discussion section.

**Comment:** As an academic paper, it is important to have a conclusion section that summaries the main objective of the study, main findings, and limitations and future work.

**Response:** Thank you for the comment. Taking this criticism into account, we added the “Conclusion” heading.

**Reviewers' comments:**

**Reviewer #1:**

**Comment:** The novelty of this paper is presented in a very limited manner. In the introduction, it is important to emphasize the significance of the work and justify its novelty by highlighting its main contributions to the existing literature.

**Response:** Thank you for the comment. Taking this criticism into account, we added the novelty of this paper.

**Comment:** The proposed hypotheses need more theoretical support.

**Response:** Thank you for the comment. Taking this criticism into account, we added additional explanations for the hypotheses.

**Comment:** Please provide a description of the population under study and the sampling technique utilized in the methodology and research design section. Additionally, please provide a rationale for the chosen sample size and sampling technique.

**Response:** Thank you for the comment. Taking this criticism into account, we added the necessary explanations to the Data section.

**Comment:** The authors are advised to dedicate a separate section to highlight the main theoretical and practical implications.

**Response:** Thank you for the comment. Taking this criticism into account, we added the “Theoretical and practical implications” heading.

**Reviewer #2:**

**Comment:** The literature review is presented in a very limited manner. Thus, it is recommended to extend the literature by including further recent, related and well-established studies. This includes but not limited to:

E-commerce in high uncertainty avoidance cultures: The driving forces of repurchase and word-of-mouth intentions. Doi:

https://doi.org/10.1016/j.techsoc.2022.102083

Feasibility assessment for E-commerce: A data collection from developing country (Ethiopia). Doi: <https://doi.org/10.1016/j.mex.2022.101639>

**Response:** Thank you for the comment. Taking this criticism into account, we have cited both the recommended studies and other new studies for literature review.

**Comment:** As e-commerce is highly researched, the discussion part can be broadened a bit more with more relevant research studies.

**Response:** Thank you for the comment. Taking this criticism into account, we revised the discussion section.

**Comment:** As an academic paper, it is important to have a conclusion section that summaries the main objective of the study, main findings, and limitations and future work.

**Response:** Thank you for the comment. Taking this criticism into account, we added the “Conclusion” heading.
